# Caregiver network roles and health and well-being among Latino older adults

**DOI:** 10.1093/geroni/igaf122.4241

**Published:** 2025-12-31

**Authors:** Nell Compernolle, Ellen Bloss, Emily Bray, Alan Tiburcio, Melissa Howe, Lissette Piedra

**Affiliations:** NORC at the University of Chicago, Chicago, Illinois, United States; NORC at the University of Chicago, Chicago, Illinois, United States; NORC at the University of Chicago, Chicago, Illinois, United States; NORC, Chicago, Illinois, United States; NORC at the University of Chicago, Chicago, Illinois, United States; University of Illinois at Urbana-Champaign, Urbana, Illinois, United States

## Abstract

Prior research links caregiving to adverse health outcomes, especially when caregivers support individuals with Alzheimer’s disease or related dementias. Importantly, studies show that diverse caregiving networks that include kin and non-kin are associated with lower caregiver burden and improved health. Using data from a new, nationally representative survey of (n = 733) Latino caregivers, we explore how specific network roles within caregiver networks impact caregiver health and wellbeing. We examine the social contexts of informal caregiving in the United States, focusing on the wellbeing of Latino caregivers of older adults. We define distinct roles within caregiver networks: care helpers (individuals who assist the caregiver in providing care), care disruptors (who hinder/complicate caregiving), and personal helpers (who support the caregiver personally, but not with caregiving). Preliminary findings indicate that kin comprise most roles - 80% of care helpers, 84% of care disruptors, and 87% of personal helpers. Caregivers with care helpers in their network report decreased feelings of loneliness (OR = 0.84, SE = 0.08, p < 0.10), while care disruptors significantly increase loneliness (OR = 1.62, SE = 0.44, p < 0.10). As the proportion of kin in care disruptor roles increases, caregivers experience even greater loneliness (OR = 1.68, SE = 0.45, p < 0.10) and depression (OR = 1.68, SE = 0.44, p < 0.05). Caregivers with personal helpers report slightly increased loneliness, depression and lower self-rated health, but results were not significant. Caregivers of individuals with ADRD reported higher loneliness (OR = 1.32, SE = 0.22, p < 0.10, depression (OR = 1.40, SE = 0.23, p < 0.05), and poorer health (OR = 1.91, SE = 0.31, p < 0.001). This study builds on frameworks situating caregiving within broader social networks by examining specific roles within Latino caregiver networks.

